# Bibliographical

**Published:** 1883-09

**Authors:** 


### Bibliographical.

*Microscopical Diagnosis;* by CHAS. H. STOWELL, M. D., and LOUISA REED STOWELL, M. S. Detroit: Geo. A. Davis, Medical Publisher. Price $3.00

This is a very handsome, large octavo volume of two hundred and fifty pages, illustrated with one hundred and twenty-eight engravings on wood, and forty-seven on stone. The authors are well known in the profession, and to microscopists generally as the editors of "The Microscope," a bi-monthly journal devoted to the use of the microscope in practical medicine and pharmacy.

The book is divided into three parts; the first, by Dr. Stowell, treating of the use of the microscope in the examination of blood, epithelium, tumors, sputa, urinary deposits, etc.

The second part, by Mrs. Stowell, is chiefly devoted to pharmacy, and the study of vegetable histology, with special reference to foods and medicinal plants.

The third department is by Mr. Wm. H. Walmsley, of Philadelphia, who is well known as an expert manipulator of the microscope, and it is devoted to the preparation and mounting of microscopical objects.

All the parts are well written, and contain information that is indispensible to the really successful practice of any department of medicine. As a work of ready reference it is especially useful, because of the profuse illustrations of microscopical tissues and deposits. It is very handsomely printed and bound, with sides and back embossed in black and gold.

